# Proximate Composition, Retained Water, and Bacterial Load for Two Sizes of Hybrid Catfish (*Ictalurus furcatus* × *Ictalurus punctatus*) Fillets at Different Process Steps

**DOI:** 10.3390/foods12051112

**Published:** 2023-03-06

**Authors:** Manirul Haque, Juan L. Silva

**Affiliations:** 1 Department of Food Science, Nutrition and Health Promotion, Mississippi State University, Mississippi State, MS 39762, USA; 2 Department of Food Science and Technology, University of Nebraska-Lincoln, Lincoln, NE 68588, USA

**Keywords:** retained water, catfish, proximate composition, water content, processing, bacterial load

## Abstract

The catfish processors in the US are required to state the maximum percentage of retained water content (RWC) on the product label. The objectives of our study were to quantify the RWC of processed hybrid catfish fillets from proximate composition and the bacterial load at different processing points. Water content was determined using oven-dry (AOAC950.46,1990) and Near-infrared (NIR) spectroscopy. Protein and fat content were determined by NIR spectrometer. Psychrotrophic (PPC) and Total Coliform (TCC) counts were enumerated using 3MPetrifilm^TM^. The fillets’ overall baseline water, protein, and fat content were 77.8, 16.7 and 5.7%, respectively. The RWC of final fresh and frozen fillets were ~1.1=/- 2.0% (not significant) and ~4.5%, respectively, and was not fillet size or harvest season dependent. Baseline water content (78.0 vs. 76.0%) was higher (*p* ≤ 0.05), and fat content (6.0% vs. 8.0%) was lower (*p* ≤ 0.05) for small (50–150 g) compared to large fillets (150–450 g). Higher (*p* ≤ 0.05) baseline PPC (~4.2 vs. ~3.0) and TCC (~3.4 vs. ~1.7) were observed for the warm season (April–July) fillets compared to the cold season (Feb–April). This study provides information to processors and others on estimating retained water and microbiological quality of the hybrid catfish fillets over the process line.

## 1. Introduction

Farm-raised catfish is a significant commercial food commodity in the United States, contributing around $4 billion to the US economy each year [1,2]. The commercial catfish processors produce the hybrid catfish for greater fillet yield [3,4]. Catfish growers directly sell food-size (0.3 to 1.5 kg) fish to processors who process them into whole fish, dressed fish, fillets, fillet strips, nuggets, and steaks [2]. These products are usually sold as either iced, frozen, battered, breaded, or fresh [5,6]. The United States Department of Agriculture-Food Safety Inspection Service (USDA-FSIS) started inspecting Siluriformes including catfish in September 2017 [7]. Fresh or fresh-frozen packages of catfish or parts must be labeled to reflect 100% net weight after thawing. Processors must state the maximum percentage of retained water (if any) on the product label [8]. The USDA-FSIS adopted existing meat and poultry net weight and retained water regulations (9 CFR Parts 381 and 441) for Siluriformes’ products (USDA, 2001) without modification. A significant number of studies reported that poultry carcasses retained 4 to 11% water after immersion chilling which may be different from catfish retained water [9,10,11,12]. Arbitrary adoption of poultry retained water regulations in the Siluriformes industry could be misleading. A data gap exists in estimating catfish retained water during and after processing. One of our goals of this study is to estimate the fresh and processed catfish (final product) retained water for filling out the existing data gap. The catfish industry also needs to identify the key variables that affect the water uptake or loss in catfish products during or after processing, which may contribute to better process control.

During commercial processing, a chilling procedure may contribute to ‘added water’ in the final product. The added water could be determined using catfish muscle water-protein ratio as protein content typically remains constant with the process steps [13,14,15]. Water content has an inverse relationship with fat content of fish [15,16,17]. This relationship could be used to predict the water content from fat or protein content of the processed catfish [11]. However, proximate composition of fish differs from species to species, individual to individual, considering size, sex, season, feeding, and processing stress [18,19,20,21]. Many studies have reported channel catfish baseline proximate composition [22,23,24], while data for hybrid catfish proximate composition in the process lines are scarce.

In a fish processing facility, indicator bacterial counts could reveal temperature abuse, cross-contamination, and mishandling during processing [21,25]. However, catfish’s bacterial counts (APC, PPC, TCC, and *E. coli*) differ with harvesting season, size of the processing plant, and processing methods [26,27]. Although some studies [26,27,28,29] reported bacterial load in the different process steps for channel catfish (*Ictalurus punctatus*) fillets; data for hybrid catfish fillets bacterial load are insufficient to control the existing catfish processing lines. The experimental data from our study will help identify the critical process steps for controlling the bacterial load in catfish fillets.

This study aimed to quantify the retained water content from the estimated proximate composition of hybrid catfish fillets and the bacterial load at different process steps. The purpose of determining the proximate composition and bacterial load at each process step was to identify the critical process step where fillets uptake the highest amount of the water content and had the highest bacterial load. Because the baseline water (natural or as received in the plant) content dictates the amount of retained water with each process step, fillet sizes and harvesting seasons were included to observe any impact of these parameters on the fillet’s water content and bacterial load as received. The natural composition, especially water content of catfish products before and during processing, can provide information to processors and inspection authorities regarding regulatory compliance and labeling requirements.

## 2. Materials and Methods

### 2.1. Sample Collection and Treatment

**Figure 1:**
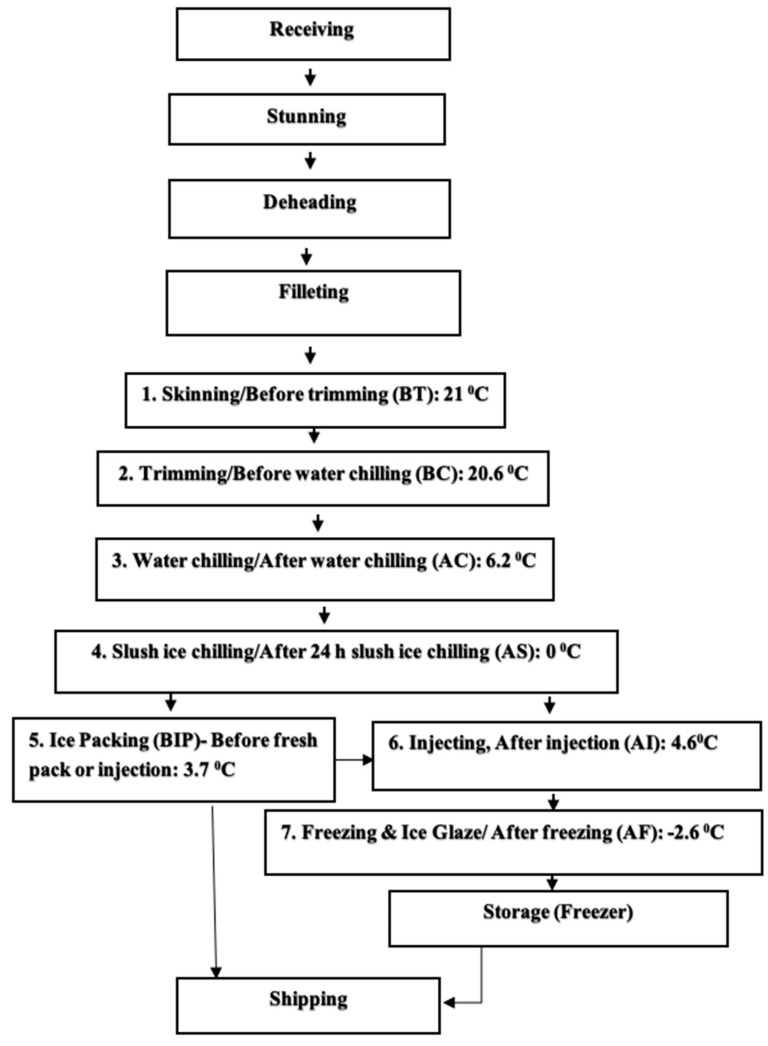
Typical process flow for catfish fillet showing sampling points.

A total of 228 hybrid catfish [Blue (*Ictalurus furcatus)* × Channel *(Ictalurus punctatus*)] fillets were collected from two local catfish processing plants in Mississippi between February to April and May to July 2018. Three fillet samples (one for microbiological analysis and two for proximate analysis) of two sizes (small: 50 g to 150 g and large: 150 g to 450 g) from seven process steps; Fillets before trimming (BT): assumed to have similar proximate composition as received fish in the processing plant, after trimming/before water chilling (BC), after water chilling (AC), after ice slush chilling (AS), before ice packing (BIP): fresh scalded fillets; fillets after injection (AI), and after freezing (AF) (Figure 1), were randomly picked and placed into quart size Ziplock^®^ bags (GreatValue^TM^ Slide Zipper 7in’8 in). The temperature of the BT, BC, AC, AS, BIP, AI, and AF fillets during sampling averaged 21 °C, 20.6 °C, 6.2 °C, 0 °C, 3.7 °C, 4.6 °C, and −2.6 °C, respectively. The sampled catfish fillets were kept in an ice chest with ice and transported within 40 min to the Food Safety and Processing Laboratory of the Department of Food Science, Nutrition and Health Promotion at Mississippi State University. Microbiological analysis was performed within six hours of sampling. Collected catfish fillets (placed in bags) kept in the ice chest covered with ice were placed at 4 °C in a refrigerator (Isotemp Plus Laboratory Refrigerator, Fisher Scientific, Pittsburg, PA, USA) for 22 to 24 h before proximate analysis.

### 2.2. Proximate Analysis

The weight and length of fillets were measured before proximate analysis. Ice glaze of the frozen fillets was removed by spraying cold water and draining the water for two minutes and immediately transferred to the refrigerator (4 °C) for further proximate analysis (AOAC 963.18). The water content was measured following AOAC 950.46,1990 procedure. In brief, the whole fillet was homogenized with a food chopper (Black & Decker^@^ Handy Chopper Plus^TM^, Towson, MD, USA) and transferred to a large (150’15 mm) Petri dish (Falcon 35 1058 PetriDish Style Sterile, Oxnard, CA, USA). An aliquot of 5 g of homogenized sample was evenly distributed into the weighing dish (Fisher Scientific, 08732101, Houston, TX, USA). Dishes (with sample) were weighed and dried at 105 ± 2 °C in an ISOTEM OVEN 300 (300 series Model 318, Fisher Scientific, Houston, TX, USA) for 5 ± 2 h or until a constant weight was achieved and were placed in a desiccator (Sanplatec Corporation, Japan) for 15 ± 5 min to cool after drying. Water content was calculated on a wet basis as follows:

(1) $$\mathrm{Water}\;\mathrm{content}=\frac{\left(W2-W3\right)*100}{W2-W1}$$

where,

W1 = weight of dish (without sample)
W2 = weight of dish (with sample) before drying
W3 = weight of dish (with sample) after drying

Proximate composition (protein, fat, collagen, and water content) of the fish fillets was analyzed on a wet basis using a Near-infrared (NIR) spectrometer (Food Scan Lab Analyzer Model 78,800, Foss Analytical, Eden Prairie, MN, USA).

### 2.3. Retained Water Calculation

A significant correlation (r = 0.90, *p* ≤ 0.05) was obtained between water determined by NIR and the oven method. To establish a prediction model, water content determined by NIR was fitted using simple linear regression. The water-protein ratio (wet basis) was calculated as follows

(2) $$\mathrm{Water}-\mathrm{protein}\;\mathrm{ratio}\;(M:P)=\frac{\mathrm{Fitted}\;\mathrm{water}\;\mathrm{content}}{\;\mathrm{protein}\;\mathrm{content}\;\mathrm{determined}\;\mathrm{by}\;\mathrm{NIR}}$$

Retained water (%) was calculated based on the water retention/loss at each point of the processing as follows:

Retained water (%) = water (%) at any process point (e.g., AC, BIP, AF)—Water (%) at baseline (BT).

### 2.4. Microbiological Analysis

A 25 g fillet sample was aseptically cut with a sterile stainless-steel knife, weighed, and placed in a stomacher bag (Nasco, Whirl-Pak, 19 × 30 cm: Fort Atkinson, WI, USA). A 225 mL of 0.1% sterilized buffer peptone water (BPW) solution (Difco, Detroit, MI, USA) was added and stomached for two minutes in a laboratory blender stomacher 400 (A. J. Seward and Co., Ltd., London, England). Dilutions were made by transferring 1 mL of the homogenate into dilution tubes with 9 mL of 0.1% sterilized peptone solution. Plating was conducted on aerobic (APC) count 3M^TM^ Petrifilm (3M Co., St. Paul, MN, USA) in duplicate, and these were incubated for 72 h at 20 ± 2 °C [27] for psychrotrophic counts (PPC). *E. coli* plates were incubated for 24 to 48 h at 35 ± 2 °C on 3M^TM^ Petrifilm *E. coli* (3M Co., St. Paul, MN, USA) in duplicate for the enumeration of *E. coli* and total coliform counts (TCC) [26,30]. According to the manufacturer’s instructions, colonies were identified and enumerated using 3M™ PetrifilmPlate Reader (3M Company, Technopath, St. Paul, MN, USA). Selected plate counting was verified by the conventional (visual) counting method.

### 2.5. Experimental Design and Statistical Analysis

Data were arranged in a 2-way factorial [2 sizes of the fillets (small: 50 to 150 g; large: 150 g to 450 g) × 7 process points] randomized complete block (RCB) design with 12 replications (blocks) based on the availability of the fillets from each process point [BT: 15 fillets (small = 7, large = 8); BC:16 fillets (small = 9, large = 6); AC = 10 fillets (small = 5, large = 5); AS: 14 fillets (small = 8, large = 6); BIP: 9 (small = 3, large = 6); AI: 9 fillets (small = 5, large = 4); AF: 7 fillets (small = 3, large = 4)]. Data were unbalanced in the blocks due to the unavailability of the fillets for some replications. The General Linear and mixed Model procedure (PROC GLMMIX) of the Statistical Analysis System (SAS studio edition, 2020) was used to examine the interaction of sizes, process steps, and blocks. There was no interaction found between size and process steps and between size, process steps and blocks. Tukey’s honest significant difference (HSD) was used for the mean separation of the measurements of the fillets (*p* ≤ 0.05). Simple linear regression (SLR) and multiple linear regression (MLR) [31] models were used to calculate the correlation of the variables. All Statistical analysis were performed using SAS universal edition (2020).

## 3. Results and Discussion

### 3.1. Proximate Composition and Microbial Load of the Hybrid Catfish Fillets by Fillet Size and Harvesting Season

The proximate composition especially water content of the receiving fillets (BT fillets) as well as microbial load dictates the final products’ water retention and microbial quality during catfish processing. However, initial proximate composition and bacterial load could differ for different sizes of fillets and harvesting season. Our study estimated the initial proximate composition and microbial load of hybrid catfish fillets and presented in Table 1.

Small-sized fillets had higher (*p* ≤ 0.05) water (78.0 vs. 76.8) and lower (*p* ≤ 0.05) fat content (6.0 vs. 8.0%) (Table 1). Conversion of water into fat as fish grows may contribute to higher fat retention content in a larger fish [16,32,33]. Silva and Ammerman (1993) [34] reported higher water content (70.8% vs. 68.1%) and lower fat content (10.8 vs. 13.2%) for small (0.3 kg) sized channel catfish (*Ictalurus punctatus*) fillets than larger ones (1.0 kg). However, protein content and bacterial load (PPC and TCC) did not differ (*p* > 0.05) between sizes.

Proximate composition and bacterial load of warm weather (May to July) fish were different (*p* ≤ 0.05) from cold weather (February to April) fish. BT fillets’ water content (77.8 vs. 75.6%) was higher (*p* ≤ 0.05), and fat content (5.8 vs. 8.7%) was lower (*p* ≤ 0.05) for those collected in the cold weather than that of the warmer weather (Table 1). Some studies [35,36] reported that low mean energy value for muscle tissue during winter are related to the lower level of fat within the muscle. Nettleton et al. (1990) also reported higher water content for fillets collected in the winter than in summer (77.4 vs. 76%). However, Robinson et al. (2001) reported no seasonal (May, October, and February) impact on the water content of large sizes (0.23 to 0.45 kg) of channel catfish (*Ictalurus punctatus*) fillets.

BT fillets’ PPC was higher (*p* ≤ 0.05) for fillets collected in the cold season compared to those from the warm season (4.2 vs. 3.0 log CFU/g); however, TCC was higher (*p* ≤ 0.05) for warmer season fillets (3.4 vs. 1.7 log CFU/g). Huang and Leung (1993) [37] reported similar PPC (2.8 to 3 log CFU/mL) and TCC (1.48 log CFU/mL) in dressed channel catfish harvested from southern Georgia during the spring season. Nunez et al. (2003) also reported higher TCC in channel catfish processed in the spring than those processed in the fall or winter. Fernandes et al., (1997) reported higher counts of *E. coli* and *S. aureus* in catfish fillets collected in summer compared to those collected in winter.

### 3.2. Water and Retained Water Content of the Hybrid Catfish Fillet at Several Process Steps

The USDA-FSIS stated that ‘retained moisture’ should be documented to provide consumers with the information necessary to make reasonable purchase decisions. In 2017, the USDA-FSIS started inspecting ‘Siluriformes’ and stated that processors must state the maximum percentage of retained water (if any) on the product label. No published study reported the retained water content for the catfish’s final commercial product. We have determined the fillets’ retained water content from the proximate composition using a systematic approach so that the authority and the catfish processors could use the data for better process control.

**Figure 2:**
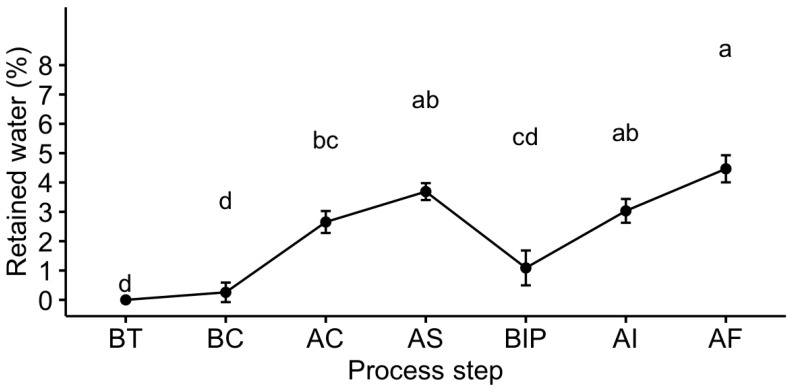
Retained water content (%) of hybrid catfish fillets at different catfish process steps regardless of size; ^abcd^ Means not followed by the same letter differ (*p* ≤ 0.05); BT = Before Trimming (Baseline; assumed to have the same proximate composition as received fish at processing plant); BC = After trimming/before chilling; AC = After water chilling; AS = After slush ice chilling; BIP = Before ice packing (Fresh fillets); AI = After injecting (polyphosphate injection), AF = After freezing (Frozen fillets).

There was no interaction (*p* > 0.05) between fillets’ sizes, harvesting season, and processing steps, indicating fillets’ sizes or seasons did not affect the retained water (water difference from baseline; BT) at any process step. Retained water was proportional to water content at every process step (Figure 2). Retained water content (RWC) differed (*p* ≤ 0.05) at some process steps. Final fresh and frozen fillets’ RWC were 1.1% with a range of 0 to 5% and 4.5% with a range of 2 to 8%, respectively (Figure 2).

Baseline (BT) retained water content (RWC) was assumed to be zero, where water content depends on the size of the fillets and seasonal changes. BC fillets’ RWC was similar (*p* > 0.05) to that of BT fillets. After water chilling, RWC for AC fillets was 2.7 ± 1.5% (with a range of 0.7 to 5%) possibly due to the adsorbance of chilled water in the subcutaneous layer of the muscle tissue [10]. After 24 h ice slush chilling, AS fillets retained more (*p* ≤ 0.05) water (3.7 ± 1.4%, with a range of 0.3 to 6.2%) than other fillets (except for AF fillets) may be due to the immersion of the fillets for a more extended period in the slush ice, where fillets trap more water in the intercellular space of the muscle tissues [10,12]. Carciofi & Laurindo (2007) [38] reported that water absorption of poultry depends on immersion time, water temperature, and water stirring conditions during chilling. Afterwards, fillets lost around 2.6% of this water content before ice packing (BIP). Retained water of the BIP fillets was 1.1 ± 2.0 with a range of −2.1 to 5.0%. Klose et al. (1960) [39] reported that most of the absorbed water is loosely held (unbound water, adsorbed) in pockets between the tissues of the muscle during immersion chilling, and most could be lost afterwards. Silva et al. (2001) supported these results stating that fillets could gain weight due to water adsorption during chilling but lose most of it before ice packing. The ranges of RWC (−0.7 to 6.3%) of hybrid catfish fillets after water and ice slush chilling were lesser than reported retained water (6 to 12%) of poultry carcass after immersion chilling [10,12]. Retained water was not different (*p* > 0.05) for fillets after injection (3.0 ± 1.6% with a range of 0.3 to 5.1%) and freezing (4.5 ± 2.0% with a range of 1.8 to 8.0%). This result might be due to the injection of polyphosphate or other chemical agents in the fillets before freezing, which increased the water binding capacity of the muscle’s myofibrillar protein and protected water loss during freezing [40,41,42].

### 3.3. Factors Affecting the Retained Water Content of Catfish Fillets

NIR spectroscopy is fast, noninvasive, and more economical for determining the proximate composition of the muscle food in comparison to other conventional (oven dry, Kjeldahl) methods [43,44]. Multiple linear regression analysis was used to analyze the factors affecting retained water of the hybrid catfish fillets during processing. Several studies [14,45] reported that the water-protein ratio could be used to determine added water during seafood processing. Breck (2014) reported that the relationship between water and protein is size-dependent, and that fat content is inversely correlated to the water content of the fish. Thus, the catfish’s water-protein ratio (M:P), weight (g), and fat content were examined by multiple linear regression analysis to predict the retained water of the catfish fillets during processing.

Water content determined by NIR spectrometer was fitted based on water content determined by oven method (AOAC approved method) using a simple linear regression model [46]. A significant correlation (F (1.74) = 513.97, *p* < 0.0001), R^2^ = 0.87) was obtained between water content determined by NIR and water content determined by the oven method. Fitted water was equal to 14.7 + 0.80 (water content determined by oven method) %.

The retained water calculated from water determined by NIR was fitted based on retained water calculated from water determined by the oven method using a simple linear regression model. The fitted retained water was equal to 3.0 + 1.10 (calculated retained water from water determined by oven) %, [(F (1.56) = 255.93, *p* < 0.0001), R^2^ = 0.82). This fitted retained water was used as a dependent variable (Y), and water-protein ratio (M:P), fat content (%), and weight (g) of the catfish were used as independent variables (X) in the prediction models. A stepwise regression analysis was conducted with backward elimination of the independent variables to fit the models. At first, all the independent variables (M:P, fat content, and weight) were used for the model establishment. The regression equation of this model (F (3, 57) = 419.36, *p* < 0.0001, R^2^ = 0.96) was as follows:

Retained water (%) = −5.6 + 2.1 (M:P) − 0.13 (Fat) + 0.0004 (weight) (Model 1)(3)

Both M:P and fat were significant (*p* ≤ 0.05) predictors for retained water; however, weight was not a significant (*p* > 0.05) predictor for retained water in this model.

Thus, weight was excluded from the model, and a reduced model (F (2, 58) = 635.59, *p* < 0.0001), R^2^ = 0.96) was established. Adjusted R-square was not different (*p* > 0.05) for this reduced model after excluding weight, indicating that weight was not a significant predictor along with water-protein ratio and fat content. The regression equation of this reduced model was as follows:

Retained water (%) = −5.6 + 2.13 (M:P) − 0.70 (Fat) (Model 2)(4)

Both M:P and fat were significant predictors of retained water in this model 2. However, when fat content was excluded from model 2, adjusted R^2^ (0.58) was different (*p* ≤ 0.05) for the reduced model 3. This indicated that fat content was a significant predictor for retained water in model 2. The regression equation of this reduced model (F (1, 59) = 84.84, *p* < 0.0001) was as follows:

Retained water (%) = −12.2 + 2.8 (M:P) (Model 3)(5)

However, when weight (g) was added excluding fat content in this reduced model 3, adjusted R^2^ (0.73) increased (*p* ≤ 0.05), which indicated that weight was a significant predictor for retained water excluding fat in model 4. The regression equation of this model (F (2, 58) = 79.78, *p* < 0.0001, R^2^ = 0.73) was as follows.

Retained water (%) = −12.3 + 3.0 (M:P) − 0.007 (weight) (Model 4)(6)

Both M:P and weight were significant predictors of model 4.

Model 2 fulfilled the goodness of fit criteria of a multiple linear regression model [46]. This model contained 76 observations and 3 parameters. The coefficient of multiple determination (R^2^) was 0.96, indicating that this model accounted for the more significant proportion of variation. The residual of M:P and fat content followed random distribution (Table 2). The value of residual degrees of freedom adjusted R square (Adj. R^2^ = 0.96) and means square error (MSE = 0.105) also exhibited a good fit of this model for the prediction of retained water based on water-protein ratio and fat content. Thus, water-protein ratio and fat content were significant predictors for retained water during processing of hybrid catfish fillets.

### 3.4. Proximate Compositions of the Hybrid Catfish Fillet at Several Process Steps 

**Figure 3:**
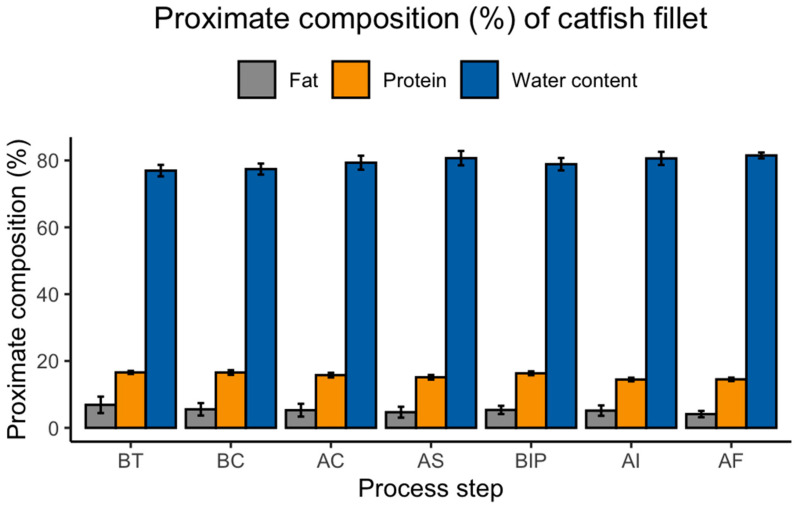
Proximate composition (%) of hybrid catfish fillets at different catfish process steps regardless of size; BT = Before Trimming (Baseline; assumed to have the same proximate composition as received fish at processing plant); BC = After trimming/before chilling; AC = After water chilling; AS = After slush ice chilling; BIP = Before ice packing (Fresh fillets); AI = After injecting (polyphosphate injection), AF = After freezing (Frozen fillets).

There was no interaction (*p ≤* 0.05) between fillet size, harvest season, and processing step on proximate composition at any process step. Fat content differed when water content differed (*p ≤* 0.05) at similar process steps (Figure 3). For instance, fillets after ice slush (AS) had less (*p ≤* 0.05) fat content in comparison to BT and BC fillets (4.7 ± 1.6 vs. 7.0 ± 2.5 and 5.6 ± 2.0%), respectively (Figure 3). An inverse correlation was reported between fat and water content of the fish fillets [15,16,17]. However, fat content of the fillets was not different (*p* > 0.05) at any of the process steps when measured on a dry basis.

Protein content was not different (*p* > 0.05) for BT (17.0 ± 0.5%), BC (17.0 ± 0.7%) and BIP (16.3 ± 0.6%) fillets (Figure 3). However, when AC and AS fillets’ water content increased (2 to 4%) due to water absorption during chilling (both water and slush ice), the percentage of protein content was lower (*p ≤* 0.05) for these fillets (AC: 16.0 ± 0.7%; AS: 15.0 ± 0.7) in comparison to BT, BC and BIP fillets. AI and AF fillets also resulted in lower (*p ≤* 0.05) protein content (AI: 14.5 ± 0.5; AF: 14.5 ± 0.5), where the water content of these (AI and AF) fillets was higher (*p* ≤ 0.05) in comparison to BT, BC and BIP fillets (Figure 3). An inverse relationship between protein and water content was also reported by Breck et al. (2014). Fillets’ protein content on a dry basis also differed (*p* ≤ 0.05) with process steps.

### 3.5. Bacterial Load of the Hybrid Catfish Fillets at Several Process Steps

**Figure 4:**
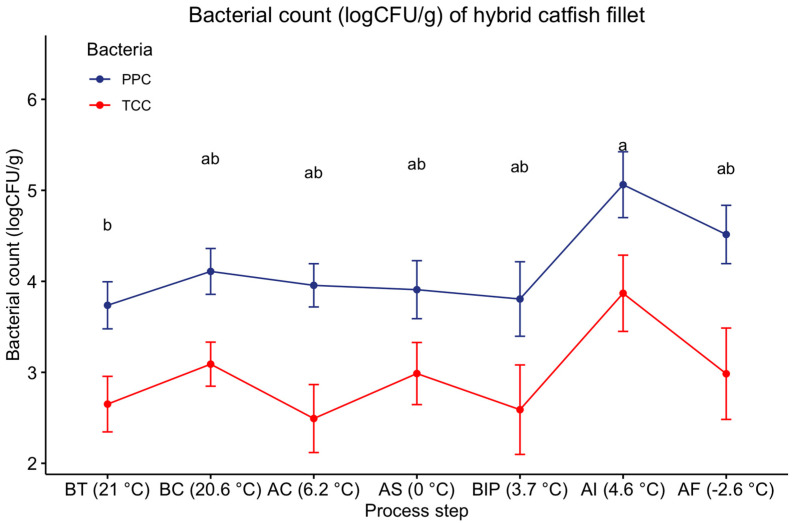
Bacterial count (log CFU/g) of hybrid catfish fillets at different process steps regardless of sizes; PPC = Psychrotrophic plate counts; TCC = Total Coliform plate counts^. ab^ Means not followed by the same letter differ (*p* ≤ 0.05); BT = Before Trimming (Baseline; assumed to have the same proximate composition as received fish at processing plant); BC = After trimming/before chilling; AC = After water chilling; AS = After slush ice chilling; BIP = Before ice packing (Fresh fillets); AI = After injecting (polyphosphate injection), AF = After freezing (Frozen fillets). Temperature in the parenthesis denotes the fillet temperature at the designated process step.

Indicator bacteria may reveal temperature abuse [47] and cross-contamination [26] during fish handling and storage. We have determined psychrotrophic plate counts (PPC) throughout the process line because of the low temperature (4.6 to 21 °C) of the processing environment where PPC could be the indicator microorganisms [29]. Total coliform plate counts (TCC) was determined to investigate cross-contamination in the processing environment. Fillets’ sizes and harvesting seasons were considered to investigate any influence of these two parameters on the final bacterial count [27]. The bacterial load data are presented in Table 1 and Figure 4.

There was no interaction (*p >* 0.05) between fillet size, harvest season, and process steps for psychrotrophic plate counts (PPC) and total coliform plate counts (TCC). The average PPC of BT fillets was ~4 log CFU/g, with a range of ND (Not detected) to ~5 log CFU/g for both sizes and seasons (Table 1). Fernandes et al. (1997) and Watchalotone et al. (1996) reported similar ranges of PPC (3.5 to 5.5 log CFU/g); however, Huang & Leung (1993) and Nunez et al. (2003) reported lower PPC (2 to 3 log CFU/g) for channel catfish (*Ictalurus punctatus*) fillets collected from processing plants. The average TCC of BT fillets was 2.4 log CFU/g, with a range of ND to ~5 log CFU/g for both sizes and seasons (Table 1).

Previous studies also reported a similar range of TCC (1 to ~2.7 log CFU/g) for channel catfish (*Ictalurus punctatus*) fillets collected from catfish processing plants [26,28,48]. The reported higher TCC results might be due to temperature abuse and mishandling, as these fillets were collected from a manual catfish processing scheme [48]. *E. coli* was not detected in any sample at any process step which could be found in other meat products [49]. The maximum acceptable limits of aerobic plate counts (APC) at 20 to 25 °C and *E. coli* in fresh and frozen fish are 5.7 log CFU/g and 1.0 log CFU/g, respectively, as specified by ICMSF (International Commission on Microbiological Specifications for Foods) [25]. However, Watchalotone et al. (2001) suggested that PPC and TCC of catfish fillets during processing should not be more than 3–4 log CFU/g and 2 log CFU/g, respectively. 

PPC and TCC of fillets were not different (*p* > 0.05) at any process steps (Figure 4) except at AI. The highest PPC and TCC was observed at this point (AI). The temperature at this step was slightly increased (~1 °C) from the previous step (BIP), which may explain the reason of higher count after injection. No differences in PPC and TCC (*p* > 0.05) for AC and AS fillets indicated that 24 h slush ice chilling could not reduce the bacterial load (PPC and TCC) in comparison to water chilling.

## 4. Conclusions

This study provided extensive data on baseline proximate composition and bacterial load of hybrid catfish fillets. As the baseline water content dictated the amount of retained water of the catfish fillets at the process steps, data (water-protein ratio and fat content) from this study could be used to estimate the fillets’ retained water at any process step which would further help the processor label their final products’ retained water. Data showed that there was no water gain in fresh fillets and minimal water gain in frozen fillets. Nevertheless, processors should be careful to estimate the retained water using baseline water content from one season to another or big fish to small ones since baseline water content is fillet size and harvest season dependent. It is worth noting that the processor should be more cautious at the injection step keeping temperature low, where the bacterial load was considerably higher than in other steps. Overall, the catfish industry and others will be benefitted using our data on estimating retained water and microbiological quality of the hybrid catfish fillets for stringent process control.

## Figures and Tables

**Table 1:** Proximate composition and bacterial count of baseline (BT) hybrid catfish fillets.

		Moisture (%) (Oven)		Fat (%) (NIR)		Protein (%) (NIR)		Psychrotrophic Counts (PPC) (log CFU/g)		Total Coliform Counts (TCC) (log CFU/g)		*E. coli*
		(mean ± SD)	Range	(mean ± SD)	Range	(mean ± SD)	Range	(mean ± SD)	Range	(mean ± SD)	Range	
**Fillet Size (Weight)**	Small (50–150 g)	78.0 ± 1.1 ^a^	76.4–80.0	6.0 ± 1.1 ^a^	4.0–7.2	17.0 ± 0.5 ^a^	16.0–17.4	3.8 ± 1.4 ^a^	0–5.4	2.3 ± 1.8 ^a^	0.0–5.4	ND
	Large (150–450 g)	76.0 ± 1.7 ^b^	73.2–78.0	8.0 ± 2.8 ^b^	4.8–12.8	16.6 ± 0.4 ^a^	15.5–17.2	3.5 ± 1.5 ^a^	0–5.5	2.6 ± 1.8 ^a^	0.0–5.5	ND
**Harvest season**	Cold (Feb–April)	77.8 ± 1.3 ^a^	74.5–80.0	5.8 ± 1.6 ^a^	4.0–10.3	16.7 ± 0.5 ^a^	15.5–17.4	4.2 ± 0.6 ^a^	3.2–5.3	1.7 ± 1.3 ^a^	0.0–3.1	ND
	Warm (April–July)	75.6 ± 1.7 ^b^	73.2–77.5	8.7 ± 2.7 ^b^	5.7–12.8	16.4 ± 0.2 ^a^	16.1–16.7	3.0 ± 2.0 ^b^	0–5.5	3.4 ± 2.0 ^b^	0.0–5.5	ND

^a,b^ Means in same column not followed by same letter differ (*p* ≤ 0.05); SD = Standard Deviation; ND = Not detected; NIR = Near infrared spectrometer method.

**Table 2:** Regression analysis of model 2 for the prediction of retained water (%) of hybrid catfish fillets during processing.

Variable	Coefficient	Std. Error	T-Statistic	Pr > |t|
Intercept	−5.734	0.590	−9.780	<0.0001
Moisture-protein ratio	2.140	0.103	20.700	<0.0001
Fat (%)	−0.670	0.030	−22.070	<0.0001
R-Squared	0.960	MSE	0.105	
Adjusted R-Squared	0.954	F-statistics	635.590	
No. of observations	61	Pr (F-statistics)	<0.0001	

Dependent variable = Fitted retained water (%) (Calculated from regression analysis between retained water from water determined by oven and NIR method; Independent variable = water-protein ratio, weight (g) and fat content (%) of the catfish fillets.

## Data Availability

Data are contained within the article.
